# Intraoperative Cortical Sensorimotor Mapping During Glioma Resection Monitored With Drum Playing During Awake Craniotomy: A Case Report

**DOI:** 10.1155/crom/4625899

**Published:** 2025-02-25

**Authors:** Priscella Asman, Israt Tasnim, Matthew Muir, Mathew Hall, Kyle Noll, Sarah Prinsloo, Giuseppe Pellizzer, Shreyas Bhavsar, Sudhakar Tummala, Nuri Ince, Sujit Prabhu

**Affiliations:** ^1^Department of Neurosurgery, The University of Texas MD Anderson Cancer Center, Houston, Texas, USA; ^2^Department of Biomedical Engineering, University of Houston, Houston, Texas, USA; ^3^Department of Neuro-Oncology, The University of Texas MD Anderson Cancer Center, Houston, Texas, USA; ^4^Research Service, Minneapolis VA Health Care System, Departments of Neurology and Neuroscience, University of Minnesota, Minneapolis, Minnesota, USA; ^5^Department of Anesthesiology and Perioperative Care, The University of Texas MD Anderson Cancer Center, Houston, Texas, USA; ^6^Department of Neurosurgery and Biomedical Engineering, Mayo Clinic, Rochester, Minnesota, USA

## Abstract

**Background:** Tumors infiltrating the precentral gyrus remain a unique operative challenge. In this study, we explored a novel approach for awake craniotomy involving a patient playing a drum pad during resection of low-grade glioma, with the use of preoperative navigated transcranial magnetic stimulation (nTMS)–generated diffusion tensor imaging (DTI) and high-density real-time electrocorticography (ECoG).

**Observation:** A 36-year-old left-handed male with a low-grade glioma in the left hemisphere hand knob region had a grand mal seizure. We combined preoperative nTMS-DTI with intraoperative passive functional mapping using high-density real-time ECoG. During an awake craniotomy, the patient played a drum pad while we assessed somatosensory-evoked potentials (SSEPs) using a 64-channel ECoG grid. This confirmed the absence of motor-evoked potentials (MEPs) over the tumor area, consistent with nTMS findings. Continuous monitoring of the patient's drum pad performance during the resection allowed for a gross total resection (GTR) of the tumor. Following the resection, he experienced some weakness in the intrinsic muscles of his right hand, which returned to full normal function at 6 months. At the end of 1 year, he remained seizure-free.

**Conclusion:** A multimodal mapping strategy combined with awake monitoring of drum playing enabled preservation of function while achieving GTR in a patient with a motor-eloquent glioma.

## 1. Introduction

To optimize tumor resection and enhance functional outcomes for patients with tumors in eloquent brain regions using awake craniotomy [1], cortical mapping before surgical resection and monitoring of patient function during the procedure are imperative to safeguard the eloquent areas and maximize patient outcome [2].

Preoperative transcranial magnetic stimulation (TMS) is a noninvasive mapping technique used to delineate functional regions of the cerebral cortex [3]. It exhibits high sensitivity and specificity in detecting cortical representations of both upper and lower extremities within the sensorimotor regions [4]. Moreover, the integration of navigated transcranial magnetic stimulation (nTMS) with diffusion tensor imaging (DTI) provides detailed and robust maps of subcortical structures [5], which has been demonstrated to be effective in predicting functional motor deficits following tumor resections in eloquent brain regions [6].

Intraoperative cortical mapping with electrocorticography (ECoG) has proven to be an efficient and safe means of identifying critical functional brain regions [7]. Previous studies have uncovered distinctive gamma (70–110 Hz) oscillatory activity in sensorimotor regions, indicating specific responses elicited by motor movements [8] and in response to somatosensory stimulation [9].

In addition to these mapping methodologies, performance-based monitoring during resection is critical to prevent loss of function. Despite this, the practice of monitoring patient-specific task performance during resection, such as musical ability, is not widely adopted [10]. Playing a musical instrument entails complex cognitive processes necessitating coordination across various brain regions beyond just the sensorimotor area [11]. This area assumes a pivotal role in planning and executing the intricate movements essential for musical proficiency [12]. Importantly, musical performance encompasses more than just fine motor skills; it also involves higher-order cognitive functions like auditory processing, timing, memory, and motor coordination. Therefore, monitoring a musical task intraoperatively, such as drumming, may offer enhanced sensitivity in detecting subtle motor and cognitive changes during resection, providing deeper insight into the cerebral mechanisms of music. This approach could surpass traditional motor monitoring techniques (e.g., grip strength or finger–thumb opposition) in terms of sensitivity and clinical relevance.

In this study, we present the case of a patient with a low-grade glioma located in the hand knob region, whose tumor was resected using an awake craniotomy. The procedure combined preoperative and intraoperative mapping with performance monitoring specifically tailored to the patient's musical abilities, highlighting the cognitive and motor demands of musical performance as a novel method for optimizing functional preservation.

## 2. Case Report

### 2.1. Initial Presentation

**Figure 1:**
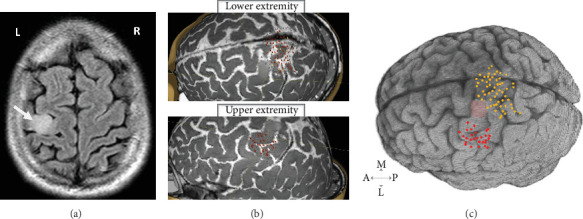
(a) Representative fluid-attenuated inversion recovery (FLAIR) MRI on admission in axial view, showing left posterior frontal lobe mass measuring 1.7 × 1.4 cm at the left precentral gyrus. L, left; R, right. (b) Preoperative nTMS-positive sites on the upper and lower extremities of the sensorimotor area superimposed on the T2-weighted MRI. Red dots represent the upper extremity and lower extremity motor-evoked potentials. (c) Coregistered nTMS points on the 3D rendering of the patient's brain. Red dots correspond to the upper extremity, and yellow dots correspond to the lower extremity. The pink shaded region shows tumor infiltration. A, anterior; P, posterior; M, medial; L, lateral.

A 36-year-old left-handed male amateur drummer presented with a grand mal seizure. Magnetic resonance imaging (MRI) revealed a mass isolated to the left hemisphere's hand knob region (Figure 1(a)). The patient was started on levetiracetam and subsequently consented to undergo an awake craniotomy. He was neurologically intact prior to surgery and provided consent for the publication of his image and case description. This study was approved by the Institutional Review Board of the University of Texas MD Anderson Cancer Center.

### 2.2. Preoperative Course

**Figure 2:**
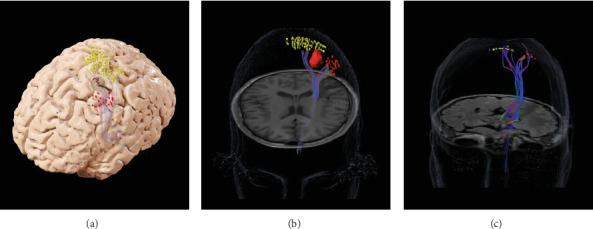
(a) Preoperative lesion volume with nTMS points shown as red dots for the upper extremity and yellow dots for the lower extremity associated with diffusion tensor imaging (DTI) tractography. (b) Preoperative DTI tractography seeded by cortical TMS points with the tumor shaded red between the tracts. (c) nTMS-seeded tractography 1 year later showing preservation of corticospinal tracts.

Preoperative nTMS data were acquired from the affected hemisphere using the Nexstim NBS4 system, equipped with a figure-of-eight double coil and integrated neuronavigation. The procedure followed a previously established pipeline [13]. During nTMS mapping, the primary sensorimotor areas for the upper and lower extremities were stimulated, with motor-evoked potentials (MEPs) recorded using surface electrodes placed on the abductor pollicis brevis for the upper extremity and on the tibialis anterior and plantar fascia for the lower extremity. Cortical excitability was assessed, revealing resting motor threshold (rMT) values of 35% of the maximum stimulator output for the upper extremities and 75% for the lower extremities. Notably, nTMS was negative within the tumor-occupied areas, while adjacent regions exhibited positive nTMS points (Figure 1(b)). Using high-resolution fluid-attenuated inversion recovery (FLAIR) imaging, we rendered the 3D volume of the patient brain and coregistered the nTMS-positive points for visualization by the neurosurgeons (Figure 1(c)). These preoperative nTMS data were used for seeding DTI tractography in BrainLAB (BrainLAB AG, Munich, Germany) as previously described [6]. This revealed DTI tracts originating from the upper and lower extremity cortical localizations, and no tracts projecting from the cortex appeared infiltrated by the tumor (Figures 2(a) and 2(b)).

### 2.3. Intraoperative Passive Sensorimotor Mapping and Surgery

The surgery was conducted using the asleep–awake–asleep method [14], coupled with intraoperative ECoG and active patient participation involving playing a drum practice pad. During the initial asleep phase, we used a 64-channel grid for ECoG data recording (5-mm intercontact space, 3-mm exposure, AdTech Medical Instruments Co., Wisconsin, United States). We employed a clinical EMG/EP Measuring System to stimulate the contralateral median nerve. Stimulation parameters were adjusted for muscle twitches. We conducted over 150 trials and recorded electromyography (EMG). Data were collected using a biosignal amplifier and synchronized in real time with Simulink/MATLAB. Somatosensory-evoked potential (SSEP) spatial–temporal features and late gamma activity were processed and displayed as a 2D heat map as previously described [15].

**Figure 3:**
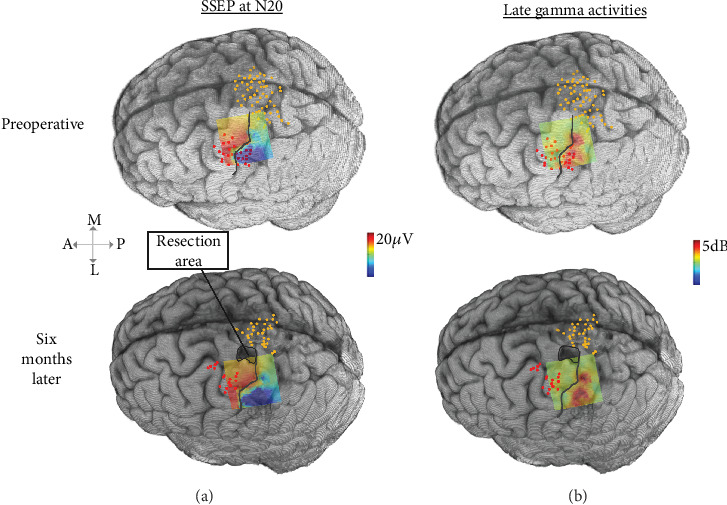
(a) ECoG SSEPs at 20 ms poststimulation as a heat map coregistered on the 3D rendering of the patient's brain. Blue indicates active somatosensory areas, while red indicates active motor areas. (b) Late gamma ECoG SSEP modulations with red areas indicating somatosensory activity. nTMS points are marked as red dots for upper extremity and yellow dots for lower extremity. A, anterior; P, posterior; M, medial; L, lateral.

**Figure 4:**
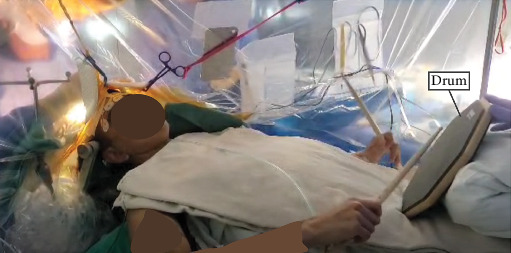
Patient playing his drum during the resection period of the awake craniotomy.

The spatial heat maps were coregistered on the 3D rendering of the patient's brain, along with the nTMS-positive points (Figure 3), and displayed for the attending neurosurgeon in the OR. The spatial–temporal activities of the SSEPs clearly showed a separation between the anterior (red) and posterior (blue) areas, with peak activities corresponding to the nTMS points of the upper extremity (Figure 3(a)). Resection margins were determined using a combination of ECoG SSEPs and tractography maps (Figure 2(b)). This information was further corroborated with anatomical landmarks identified from preoperative imaging, utilizing Brainlab intraoperative navigation for precise guidance. Upon patient awakening, SSEP recordings were repeated, and the spatial–spectral features of the SSEP defined the primary somatosensory areas, with peak (red) activity aligned with the nTMS points indicating strong spatial consistency (Figure 3(b)). The patient played the drum throughout the resection to monitor for any changes in motor function (Figure 4). Towards the end of the resection, he developed some weakness in the intrinsic muscles and wrist extensors of his right hand which altered the playing rhythm. At this time, resection was concluded with a safe-maximal resection of the tumor achieved based on the FLAIR hyperintensity visualized in the neuronavigation system and margins from intraoperative ultrasound. A video of the case where the subject was playing the drums is provided in the supporting information (Video S1).

### 2.4. Postoperative Course

**Figure 5:**
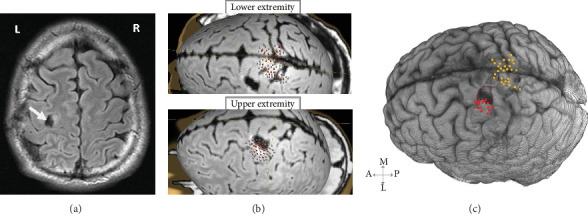
(a) Postoperative axial FLAIR MRI 1 year later in axial view showing resection cavity. L, left; R, right. (b) Follow-up nTMS 1 year later superimposed on FLAIR MRI. Red dots represent points with MEPs for upper and lower extremity. (c) 3D rendering of the patient's MRI 1 year later with the nTMS points coregistered. Red dots represent the upper extremity, and yellow dots represent the lower extremity. A, anterior; P, posterior; M, medial; L, lateral.

Immediately after surgery, MRI confirmed gross total resection (GTR) of the tumor and the patient exhibited some early postoperative weakness (MRC Grade 4/5) in the right wrist extensors and hand intrinsics. At the 6-month follow-up, he had improved hand function and was fully functional, and the preoperative nTMS sites and corresponding intraoperative ECoG spatial–temporal and spatial–spectral SSEP hotspots (Figure 3) were confirmed as entirely preserved. Repeat nTMS conducted at this follow-up showed preserved motor functions, with a rMT of 40% for the upper extremity and 77% for the lower extremity. A 1-year follow-up showed stable MRI scans (Figure 5(a)) and no reported interval seizures. Repeat nTMS sensorimotor mapping (Figure 5(b)) showed consistent rMT values of 40% for the upper extremity and 75% for the lower extremity. Additionally, nTMS-seeded DTI tractography confirmed the preservation of corticospinal tracts (Figure 2(c)). The tumor resection area remained negative (Figure 5(c)), and the patient maintained his baseline drumming ability. The pathology was consistent with a WHO Grade II astrocytoma, IDH-mutant, and the plan is to continue to follow the patient without any adjuvant treatments.

## 3. Discussion

We present a novel approach to resecting a low-grade glioma located in the motor cortex that involves monitoring motor function during an awake craniotomy by having a patient play a drum pad in the operating room, along with both preoperative and intraoperative multimodal mapping of the sensorimotor areas. The utility of this method is evidenced by the successful tumor resection and resolution of seizures, without any observed decline in the patient's performance after the surgery. Importantly, the real-time intraoperative change in musical function alerted the surgeon to stop the resection in a timely manner. Further, this relatively subtle alteration in function would have been unobserved with more traditional monitoring of gross motor strength and basic dexterity.

Preoperatively, nTMS successfully identified active sensorimotor areas surrounding the tumor in the hand knob region, and DTI tractography seeded by nTMS results confirmed a clear separation between the tumor and the corticospinal tract. Intraoperatively, real-time ECoG mapping with SSEPs provided a precise delineation of the central sulcus. Our approach, based on prior work, demonstrated that utilizing the spatial, spectral, and temporal features of SSEPs in real time on a high-density grid enables accurate spatial delineation of sensorimotor eloquent regions [15, 16]. This case also lends support to our prior work in which preservation of nTMS-informed DTI motor tracts prevents permanent postoperative motor deficits [6]. Notably, consistent rMT values were observed at the 6-month and 1-year follow-ups, remaining comparable to preoperative levels and within the healthy range [17].

In contrast to engaging patients in conversation or having them perform routine simple hand movements [18, 19], real-time intraoperative drum playing represents a real-world skill important to patient quality of life involving complex coordination of sensorimotor functions. The patient's drum playing provided excellent feedback as demonstrated by a noticeable change in rhythm when the patient developed some weakness, providing the surgeon further confidence in the margins necessary for safe resection and assurance to the patient that hand functionality was not critically diminished.

Currently, there is limited experience in using musical instruments for intraoperative patient assessment during tumor resection, aside from a few reports regarding positive outcomes in patients that played clarinet [20], violin [21],, and guitar [10, 22, 23] in the intraoperative setting. Piai et al. [21] documented a case where a patient's skill in playing the violin during surgery allowed for the detection of impairment, leading to a pause in the resection and the prevention of neurological deterioration. Similarly, Horisawa et al. [23] reported that playing the guitar and violin during surgery for musician's dystonia facilitated precise symptom monitoring. Scerrati et al. [22] emphasized that playing the clarinet in the operating room engages higher cognitive functions more effectively than simpler tasks. Mackel, Orrego-Gonzalez, and Vega [10] found that having a patient play the guitar during surgery can not only preserve but also enhance their musical abilities postsurgery through improvement in preoperative deficits. Furthermore, several studies have highlighted that this approach helps identify and preserve specific cortical areas related to music performance during surgery [22, 24, 25].

This case report represents the first instance of a drum being used to assess hand and wrist function during a craniotomy for tumor resection. Drumming especially engages both gross and fine motor skills and cognitive–motor rhythmic synchrony through complex coordination and precise timing, making it particularly effective for detecting subtle changes in motor function. The immediacy and visibility of rhythm changes can provide rapid feedback to the surgical team, allowing for real-time adjustments to approach. Compared to simple finger tasks, drumming also involves a higher level of motor complexity and cognitive processing, offering a more comprehensive assessment of neurological function and sensorimotor network integrity. Additionally, the broader motor engagement and accessibility of drumming make it a versatile and sensitive tool for intraoperative neurological assessment, offering a promising method for monitoring complex motor abilities in the hand and wrist. This may represent a model for more fine-grained and sensitive intraoperative monitoring of motor abilities, even in patients without concerns regarding the preservation of musical abilities. That is, incorporating more complex motor tasks with varying speeds and rhythmic demands could improve intraoperative monitoring, helping to preserve motor functions essential for the patient's daily activities and quality of life.

## 4. Conclusion

In this case study, integrating advanced preoperative mapping techniques, nTMS and DTI, with real-time intraoperative mapping and monitoring through ECoG and musical task performance is a feasible approach for preserving musical ability in patients undergoing tumor resections in motor-eloquent brain regions. This comprehensive approach allows for the precise identification and preservation of critical motor and cognitive functions essential for musical performance. Although the findings are based on a single case without a control group, the methodology offers valuable insight into the potential for tailored functional monitoring during surgery, particularly in patients with unique motor and cognitive demands, such as musicians.

## Data Availability

The data supporting the findings of this study are available upon request from the corresponding author. However, the data is not publicly accessible due to privacy and ethical restrictions.

## Supporting Information

Supporting Information Additional supporting information can be found online in the Supporting Information section. The supporting information includes a video showing the intraoperative setup where the patient plays a drum pad while the tumor is being resected. Initially, the patient exhibits normal hand function; however, as the resection progresses, noticeable impairments in hand function begin to appear. This material is significant because it clearly demonstrates how the patient's hand function visibly changes as he continues to play the drum, capturing nuances that standard monitoring methods, such as SSEP, might not easily detect.

## References

[1] Clavreul A., Aubin G., Delion M., Lemée J. M., Ter Minassian A., Menei P. (2021). What effects does awake craniotomy have on functional and survival outcomes for glioblastoma patients?. *Journal of Neuro-Oncology*.

[2] Kim S. S., McCutcheon I. E., Suki D. (2009). Awake craniotomy for brain tumors near eloquent cortex: correlation of intraoperative cortical mapping with neurological outcomes in 309 consecutive patients. *Neurosurgery*.

[3] Weise K., Numssen O., Kalloch B. (2023). Precise motor mapping with transcranial magnetic stimulation. *Nature Protocols*.

[4] Rosenstock T., Tuncer M. S., Münch M. R., Vajkoczy P., Picht T., Faust K. (2021). Preoperative nTMS and intraoperative neurophysiology-a comparative analysis in patients with motor-eloquent glioma. *Frontiers in Oncology*.

[5] Ohue S., Kohno S., Inoue A. (2012). Accuracy of diffusion tensor magnetic resonance imaging-based tractography for surgery of gliomas near the pyramidal tract: a significant correlation between subcortical electrical stimulation and postoperative tractography. *Neurosurgery*.

[6] Muir M., Prinsloo S., Michener H. (2022). TMS seeded diffusion tensor imaging tractography predicts permanent neurological deficits. *Cancers (Basel)*.

[7] Wang Y., Fifer M. S., Flinker A. (2016). Spatial-temporal functional mapping of language at the bedside with electrocorticography. *Neurology*.

[8] Jiang T., Pellizzer G., Asman P. (2020). Power modulations of ECoG alpha/beta and gamma bands correlate with time-derivative of force during hand grasp. *Frontiers in Neuroscience*.

[9] Ray S., Niebur E., Hsiao S. S., Sinai A., Crone N. E. (2008). High-frequency gamma activity (80–150Hz) is increased in human cortex during selective attention. *Clinical Neurophysiology*.

[10] Mackel C. E., Orrego-Gonzalez E. E., Vega R. A. (2023). Awake craniotomy and intraoperative musical performance for brain tumor surgery: case report and literature review. *Brain Tumor Research and Treatment*.

[11] Schwenkreis P., El Tom S., Ragert P., Pleger B., Tegenthoff M., Dinse H. R. (2007). Assessment of sensorimotor cortical representation asymmetries and motor skills in violin players. *European Journal of Neuroscience*.

[12] Bizzi E., Ajemian R. (2020). From motor planning to execution: a sensorimotor loop perspective. *Journal of Neurophysiology*.

[13] Muir M., Prinsloo S., Michener H. (2022). Transcranial magnetic stimulation (TMS) seeded tractography provides superior prediction of eloquence compared to anatomic seeded tractography. *Neuro-Oncology Advances*.

[14] Huncke K., Van de Wiele B., Fried I., Rubinstein E. H. (1998). The asleep-awake-asleep anesthetic technique for intraoperative language mapping. *Neurosurgery*.

[15] Asman P., Prabhu S., Tummala S., Ince N. F. Real-time delineation of the central sulcus with the spatial profile of SSEPs captured with high-density Ecog grid.

[16] Asman P., Pellizzer G., Tummala S. (2023). Long-latency gamma modulation after median nerve stimulation delineates the central sulcus and contrasts the states of consciousness. *Clinical Neurophysiology*.

[17] Veldema J., Nowak D. A., Gharabaghi A. (2021). Resting motor threshold in the course of hand motor recovery after stroke: a systematic review. *Journal of NeuroEngineering and Rehabilitation*.

[18] Whittle I. R., Midgley S., Georges H., Pringle A. M., Taylor R. (2005). Patient perceptions of “awake” brain tumour surgery. *Acta Neurochirurgica*.

[19] Potters J. W., Klimek M. (2015). Awake craniotomy. *Current Opinion in Anaesthesiology*.

[20] Scerrati A., Mongardi L., Cavallo M. A. (2021). Awake surgery for skills preservation during a sensory area tumor resection in a clarinet player. *Acta Neurologica Belgica*.

[21] Piai V., Vos S. H., Idelberger R., Gans P., Doorduin J., Ter Laan M. (2019). Awake surgery for a violin player: monitoring motor and music performance, a case report. *Archives of Clinical Neuropsychology*.

[22] Leonard M. K., Desai M., Hungate D. (2019). Direct cortical stimulation of inferior frontal cortex disrupts both speech and music production in highly trained musicians. *Cognitive Neuropsychology*.

[23] Horisawa S., Taira T., Goto S., Ochiai T., Nakajima T. (2013). Long-term improvement of musician's dystonia after stereotactic ventro-oral thalamotomy. *Annals of Neurology*.

[24] Dziedzic T. A., Bala A., Podgórska A., Piwowarska J., Marchel A. (2021). Awake intraoperative mapping to identify cortical regions related to music performance: technical note. *Journal of Clinical Neuroscience*.

[25] Bass D. I., Shurtleff H., Warner M. (2021). Awake mapping of the auditory cortex during tumor resection in an aspiring musical performer: a case report. *Pediatric Neurosurgery*.

