# Supplementary material for: Intraoperative Cortical Sensorimotor Mapping During Glioma Resection Monitored With Drum Playing During Awake Craniotomy: A Case Report
Source: Case Rep Oncol Med. 2025 Feb 25;2025:4625899. doi: 10.1155/crom/4625899 (PMC11879599; doi:10.1155/crom/4625899)
Supplement: Supporting Information — Additional supporting information can be found online in the Supporting Information section. The supporting information includes a video showing the intraoperative setup where the patient plays a drum pad while the tumor is being resected. Initially, the patient exhibits normal hand function; however, as the resection progresses, noticeable impairments in hand function begin to appear. This material is significant because it clearly demonstrates how the patient's hand function visibly changes as he continues to play the drum, capturing nuances that standard monitoring methods, such as SSEP, might not easily detect. [file 4625899.f1.docx]

SUPPLEMENTARY MATERIAL

**Supplementary Video 1:** A video showing the patient playing the drum pad during the awake portion of the procedure to facilitate safe and maximum tumor resection.

Link to video:

1. [Video1.mp4](https://mdandersonorg-my.sharepoint.com/personal/pramadoss_mdanderson_org/Documents/Documents/Neurosurgery/Dr.%20Prabhu/Operative%20Neurosurgery%20Case%20Report/Operative%20Neurosurgery%20Submitted%20files/Video1.mp4?csf=1&web=1&e=HGYVm6&nav=eyJyZWZlcnJhbEluZm8iOnsicmVmZXJyYWxBcHAiOiJTdHJlYW1XZWJBcHAiLCJyZWZlcnJhbFZpZXciOiJTaGFyZURpYWxvZy1MaW5rIiwicmVmZXJyYWxBcHBQbGF0Zm9ybSI6IldlYiIsInJlZmVycmFsTW9kZSI6InZpZXcifX0%3D)
2. <https://mdandersonorg-my.sharepoint.com/personal/pramadoss_mdanderson_org/_layouts/15/stream.aspx?id=%2Fpersonal%2Fpramadoss%5Fmdanderson%5Forg%2FDocuments%2FDocuments%2FNeurosurgery%2FDr%2E%20Prabhu%2FOperative%20Neurosurgery%20Case%20Report%2FOperative%20Neurosurgery%20Submitted%20files%2FVideo1%2Emp4&referrer=StreamWebApp%2EWeb&referrerScenario=AddressBarCopied%2Eview%2Edcc79c97%2D88b7%2D47d9%2D8d74%2D2e29c9f562b7>
